# Microbially Produced Imidazole Propionate Is Associated With Heart Failure and Mortality

**DOI:** 10.1016/j.jchf.2023.03.008

**Published:** 2023-04-26

**Authors:** Antonio Molinaro, Ina Nemet, Pierre Bel Lassen, Rima Chakaroun, Trine Nielsen, Judith Aron-Wisnewsky, Per-Olof Bergh, Lin Li, Marcus Henricsson, Lars Køber, Richard Isnard, Gerard Helft, Michael Stumvoll, Oluf Pedersen, J. Gustav Smith, W.H. Wilson Tang, Karine Clément, Stanley L. Hazen, Fredrik Bäckhed

**Affiliations:** a Wallenberg Laboratory, Department of Molecular and Clinical Medicine and Sahlgrenska Center for Cardiovascular and Metabolic Research, University of Gothenburg, Gothenburg, Sweden; b Sahlgrenska University Hospital, Department of Medicine, Gothenburg, Sweden; c Department of Cardiovascular and Metabolic Sciences, Lerner Research Institute, Cleveland, Ohio, USA; d Center for Microbiome and Human Health, Cleveland Clinic, Cleveland, Ohio, USA; e Sorbonne Université, INSERM, Nutrition and Obesities: Systemic Approaches (NutriOmics), Paris, France; f Assistance Publique Hôpitaux de Paris, Pitie-Salpêtrière Hospital, Nutrition Department, Paris, France; g Medical Department III-Endocrinology, Nephrology, Rheumatology, University of Leipzig Medical Center, Leipzig, Germany; h Novo Nordisk Foundation Center for Basic Metabolic Research, Faculty of Health and Medical Sciences, University of Copenhagen, Copenhagen, Denmark; i Department of Cardiology, Rigshospialet, Copenhagen University Hospital, Copenhagen, Denmark; j Assistance Publique Hôpitaux de Paris, Pitié-Salpêtrière Hospital, Cardiology Department, Paris, France; k Sorbonne Université, INSERM UMRS1166, Hôpital Pitié-Salpêtrière (AP-HP), Paris, France;; l Department of Cardiology, Clinical Sciences, Lund University and Skåne University Hospital, Lund, Sweden; m Wallenberg Center for Molecular Medicine and Lund University Diabetes Center, Lund University, Lund, Sweden; n Department of Cardiovascular Medicine, Heart, Vascular and Thoracic Institute, Cleveland Clinic, Cleveland, Ohio, USA; o Region Västra Götaland, Sahlgrenska University Hospital, Department of Clinical Physiology, Gothenburg, Sweden.

**Keywords:** heart failure, histidine, imidazole propionate, microbiota

## Abstract

**BACKGROUND:**

Over the past years, it has become clear that the microbial ecosystem in the gut has a profound capacity to interact with the host through the production of a wide range of bioactive metabolites. The microbially produced metabolite imidazole propionate (ImP) is clinically and mechanistically linked with insulin resistance and type 2 diabetes, but it is unclear how ImP is associated with heart failure.

**OBJECTIVES:**

The authors aimed to explore whether ImP is associated with heart failure and mortality.

**METHODS:**

ImP serum measurements in 2 large and independent clinical cohorts of patients (European [n = 1,985] and North American [n = 2,155]) with a range of severity of cardiovascular disease including heart failure. Univariate and multivariate Cox regression analyses were performed to delineate the impact of ImP on 5-year mortality in the North American cohort, independent of other covariates.

**RESULTS:**

ImP is independently associated with reduced ejection fraction and heart failure in both cohorts, even after adjusting for traditional risk factors. Elevated ImP was a significant independent predictor of 5-year mortality (for the highest quartile, adjusted HR: 1.85 [95% CI: 1.20–2.88]; *P* < 0.01).

**CONCLUSIONS:**

The gut microbial metabolite ImP is increased in individuals with heart failure and is a predictor of overall survival.

## CENTRAL ILLUSTRATION

**Imidazole Propionate Is Associated With Heart Failure and Survival in Individuals With Cardiovascular Disease** Gut microbiota produces imidazole propionate from histidine and individuals with high levels of imidazole propionate (Q4) have reduced survival.

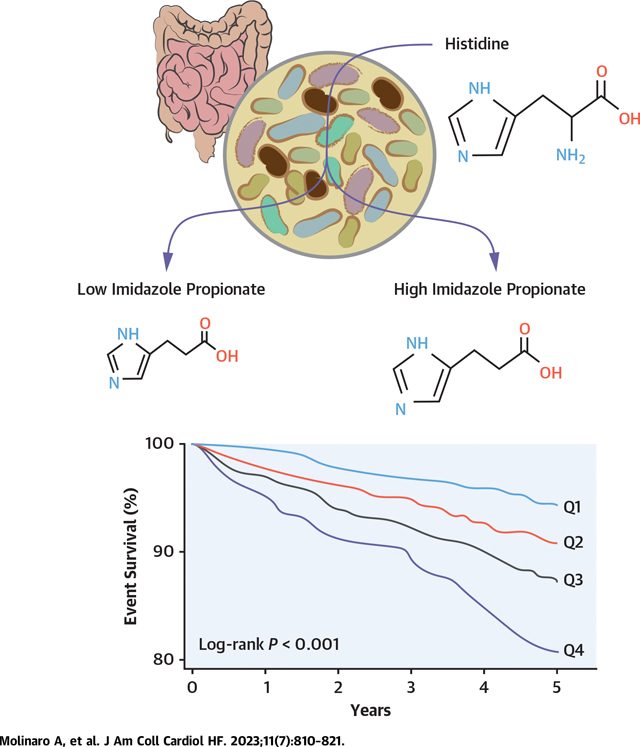

Accumulating evidence suggests that human gut microbiota can contribute to both metabolic and cardiovascular diseases (CVDs) such as coronary artery disease (CAD) and heart failure (HF).^1,2^ Metagenomic analyses of different cohorts of patients with CVDs have demonstrated altered gut microbiota composition in the presence vs absence of CVD and HF.^3–5^ Furthermore, both clinical studies with metabolomics data^6,7^ and mechanistic studies from animal models^8^ have demonstrated a potential causal role of microbiota in CVD and HF.^9–12^

Gut microbiota act at the intersection between host and diet, producing numerous metabolites that can be absorbed into the systemic circulation, where some are further modified by host enzymes and contribute to cardiometabolic diseases.^2,13,14^ These include metabolites from amino acids (eg, phenylacetylglutamine,^15^ indoxyl sulfate,^16,17^ p-cresol sulfate^16^), biogenic amines (eg, trimethylamine N-oxide [TMAO]^8,10,18^), indigestible carbohydrates (eg, short-chain fatty acids^19^), and bile acids.^20^ TMAO is produced from microbial metabolism of phosphatidylcholine,^8^ choline,^8^ or L-carnitine,^10,21^ which generates trimethylamine that is further oxidized to TMAO in the liver by flavin-containing monooxygenase 3 enzyme.^22^ TMAO has been associated with CVD,^2^ plaque vulnerability,^10,23^ atherosclerosis,^8,10,23^ thrombosis,^24–26^ stroke,^12,27^ and HF.^28,29^

We recently demonstrated that gut microbiota in individuals with insulin resistance and type 2 diabetes (T2D) has increased capacity to produce imidazole propionate (ImP) from the amino acid histidine.^30,31^ The increased production of ImP is associated with altered gut microbiota^32,33^ and bacteria that are more abundant in patients with T2D such as *Clostridium bolteae, Clostridium symbiosum*, and *Ruminococcus gnavus.*^31,34^ Furthermore, treatment of mice with ImP, or stimulation of different cell types with ImP in vitro induced activation of p38γ and subsequent activation of 2 distinct downstream signaling pathways, p62-mTORC1-S6K1^30^ and AKT-AMPK,^35^ leading to insulin resistance and lack of response to metformin, respectively. Activation of mTORC1 signaling has been implicated in the onset of CVDs and HF,^36–38^ and both p38γ/δ-mTOR and AMPK regulation are involved in heart remodeling in murine models of HF.^39–41^ However, it remains unclear if ImP is associated with HF in humans. Here we analyzed circulating ImP levels in 2 independent multicenter cohorts (1 European and 1 North American) to examine the association between ImP levels with both HF and risk of incident mortality during follow-up.

## METHODS

### STUDY POPULATIONS.

#### European cohort.

Healthy persons and those with different severity of CVDs were recruited between 2013 and 2015 in clinical institutions in France (Pitié-Salpêtrière Hospital, Center of Research for Clinical Nutrition, Institute of Cardiometabolism and Nutrition), Germany (Integrated Research and Treatment Center Adiposity Diseases in Leipzig), and Denmark (Novo Nordisk Foundation Center for Basic Metabolic Research in Copenhagen) for the European project MetaCardis.^42^ Presence of CVD was defined by clinical and imaging signs of acute myocardial infarction or chronic CAD (for more than 12 months) upon cardiac ultrasound without symptoms of HF. Presence of HF was defined upon cardiac ultrasound with a left ventricular ejection fraction (LVEF) <45% or clinical symptoms of HF from the stage 2 to 4 of the NYHA functional classification after an evaluation by a cardiologist or recent acute HF episode. As one of the aims of this cohort was to investigate metabolic diseases, the group without CVD was enriched in patients with metabolic disease such as obesity and T2D. However, a subgroup of healthy control subjects with no signs of either CVD or metabolic disease were also recruited through advertisement and through existing population cohorts. T2D was defined according to the American Diabetes Association definitions.^43^ In total, 1,985 subjects for whom serum samples were available were included for ImP analyses of which 1 clear outlier for ImP levels according to Grubb’s test was excluded along with 6 who had incomplete biochemistry data.

Patients with histories of abdominal surgery (other than appendicitis or cholecystectomy), abdominal radiotherapy, or digestive cancer or who had received recent antibiotic treatment (<2 months) were not included. All participants provided written informed consent, and the METACARDIS (Metagenomics in Cardiometabolic Diseases; NCT02059538) study was conducted in accordance with the Helsinki Declaration. The Ethics Committee of each participating country approved the clinical investigation. A detailed list of prescribed medications, anthropometric data, clinical history, and a fasting blood sample were obtained at enrollment.

#### North American cohort.

Patient samples and clinical data used for the independent validation cohort from North America were obtained from the GATC (GeneBank at the Cleveland Clinic: Molecular Determinants of Coronary Artery Disease; NCT00590200) study. GeneBank is a single-site sample repository generated from consecutive patients undergoing elective diagnostic coronary angiography or elective cardiac computed tomographic angiography with extensive clinical and laboratory characterization and longitudinal follow-up. Subject recruitment occurred between 2001 and 2007. Ethnicity was self-reported, and information regarding demographics, medical history, and medication use was obtained by patient interviews and confirmed by chart reviews. All clinical outcome data were verified by source documentation. Exclusion criteria for GeneBank included patients with recent myocardial infarctions (<4 weeks) or elevated troponin I (>0.03 mg/dL) at enrollment. CVDs were clinically defined as having previous history of documented CAD, peripheral artery disease, cerebral vascular disease (history of a transient ischemic attack or cerebrovascular accident), history of revascularization (coronary artery bypass graft, angioplasty, or stent), or significant angiographic evidence of CAD (≥50% stenosis) in at least 1 major coronary artery at the time of coronary angiography. Subjects with CAD were defined as patients with adjudicated diagnoses of stable or unstable angina, myocardial infarction, history of coronary revascularization, or angiographic evidence of ≥50% stenosis of at least 1 major coronary artery. Peripheral artery disease was defined as any clinical evidence of extracoronary vascular disease. History of HF was detected by directly asking patient by research personnel; reviewing medical records for confirmation (all patients were seen by cardiologists at Cleveland Clinic before the left heart catheterization); and International Classification of Disease (ICD) codes and adjudication by research personnel.^44^ All clinical study protocols and informed consent for human subjects were approved by the Cleveland Clinic Institutional Review Board. Written informed consent was obtained from all individuals.

### BIOCHEMICAL ANALYSES.

Blood samples were collected after an overnight fast. For the European cohort: plasma-serum samples were stored at the clinical centers at −80° C until shipment to a central laboratory facility. Fasting plasma glucose, total and high-density lipoprotein (HDL) cholesterol, triglycerides, creatinine and hemoglobin (Hb) A1c levels were measured using standard enzymatic methods. Low-density lipoprotein (LDL) cholesterol concentrations were measured enzymatically for German participants or by the Friedewald equation for French and Danish participants. N-terminal pro–B-type natriuretic peptide (NT-proBNP) and pro-atrial natriuretic peptide (proANP) were both measured centrally at a reference center in Denmark for all participants in the European cohort. The measuring laboratory supplied the consortium with batch-corrected measurements. NT-proBNP levels were measured in all GeneBank samples by the PRL (Preventive Research Lab), a CAP (College of American Pathologists) and CLIA (Clinical Laboratory Improvement Amendments) reference laboratory. Measurements for NT-proBNP were completed using the Elecsys proBNP II STAT assay on the Roche Cobas e601 analyzer (Roche Diagnostics).

Serum levels of ImP were quantified using ultra performance liquid chromatography (UPLC) coupled to tandem mass spectrometry (LC-MS/MS). Analyses were performed in 2 different laboratories in Sweden (European cohort) and in the United States (North American cohort). Both laboratories have used the same internal standard (IS) (^13^C_3_-ImP) and fully validated LC-MS/MS methods. The European cohort was analyzed based on a previously published method.^30^

North American samples (3 μL) were subjected to the LC-MS/MS analysis on a system consisting of 2 Shimadzu LC-30 AD pumps (Nexera X2), a CTO 20AC oven operating at 30° C, and a SIL-30 AC-MP auto-sampler in tandem with 8050 triple quadruple MS (Shimadzu Scientific Instruments, Inc). The limit of quantification (LOQ) 10:1 signal to noise cutoff was 5 nM. Values below LOQ were reported as one-half of the LOQ value. Three quality control samples were run with each batch of samples, and interbatch variations expressed as coefficient of variation (CV) were ≤10%. For data analysis software Lab Solution (version 5.89, Shimadzu Scientific Instruments, Inc) was used.

### STATISTICAL ANALYSIS.

**FIGURE 1:**
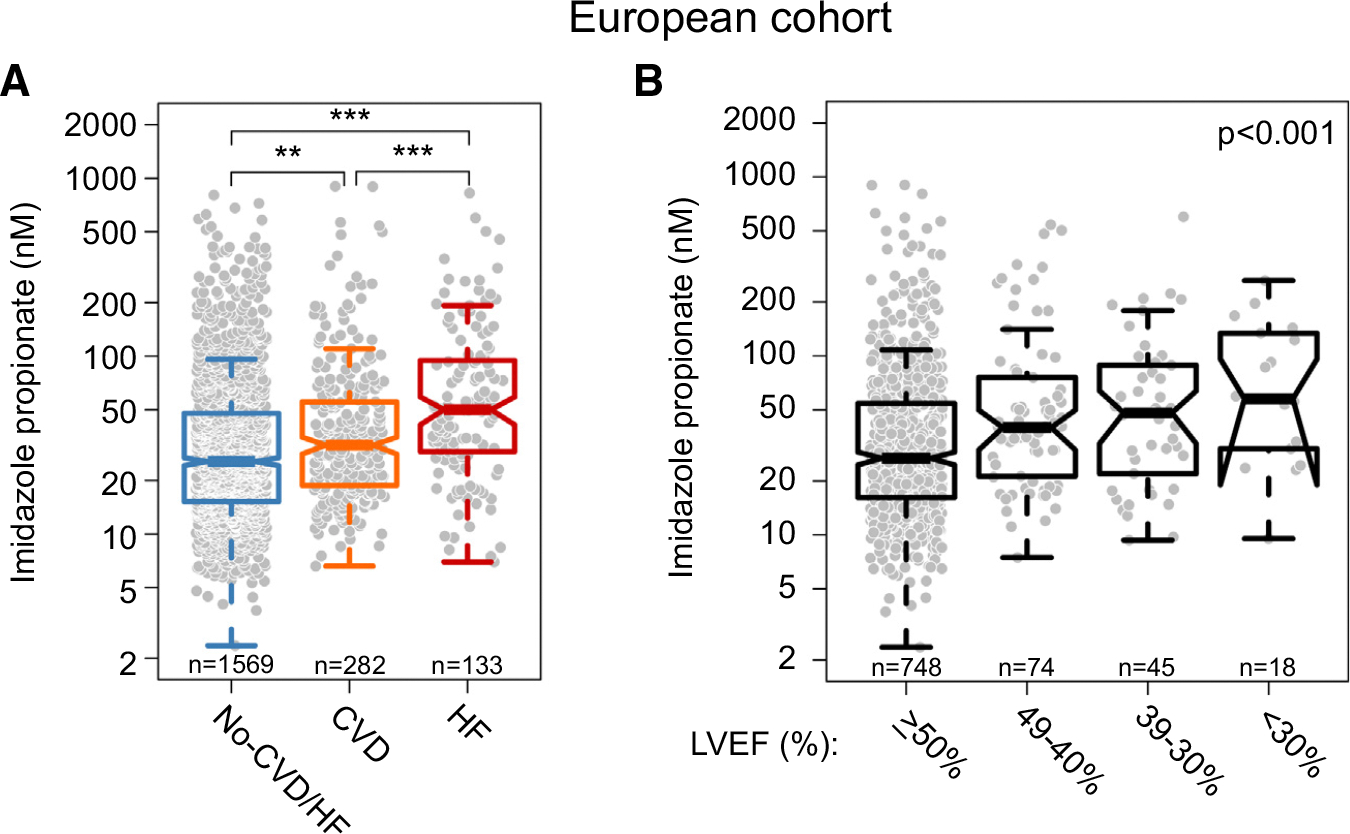
Imidazole Propionate in Individuals With Cardiovascular Diseases in the European Cohort **(A)** Serum levels of imidazole propionate in individuals without CVD and HF (n = 1,569), with CVD (n = 282), and with HF (n = 134). *P* values were calculated using Kruskal-Wallis H test, **P < 0.01, ***P < 0.001. **(B)** Serum levels of imidazole propionate according to percentage of LVEF; *P* values were calculated with linear regression model after adjustment for risk factors and other baseline covariates: age, sex, BMI, ethnicity, diabetes status, smoking status, systolic blood pressure, use of statins, serum levels of HDL, LDL, triglycerides, and eGFR). BMI = body mass index; CVD = cardiovascular disease; eGFR = estimated glomerular filtration rate; HDL = high-density lipoprotein; HF = heart failure; LDL = low-density lipoprotein; LVEF = left ventricular ejection fraction.

Estimated glomerular filtration rate (eGFR) was calculated using the Modification of Diet in Renal Disease formula.^45^ For descriptive statistics, continuous variables were presented either as median (IQR) or mean ± SD. ImP levels were categorized into quartiles in the full analysis cohorts. Categorical variables were presented as total observations and percent. Group comparisons were performed using Mann-Whitney U-test or Kruskal-Wallis H test-linear regression model, depending on whether 2 or more groups were compared, unadjusted, or adjusted for cardiovascular risk factors when appropriate (Figure 1). Odds ratio (OR) calculations were performed using binary and multinominal logistic regression models as unadjusted or adjusted for cardiovascular risk factors when appropriate (see Supplemental Tables 1 to 5). Cox proportional-hazards regression analysis for overall survival was performed unadjusted and after adjustment for cardiovascular risk factors (age; sex; body mass index [BMI]; ethnicity; diabetes status; smoking status; systolic blood pressure; use of statins; serum levels of HDL, LDL, triglycerides, and eGFR).

Variables with skewed distributions were logarithmically transformed before entering the models (Shapiro-Wilk test *P* < 0.05). Statistical analyses were carried out using IBM SPSS v26 and R statistical analysis software version 3.3.2 (IBM Corp).

Cox proportional hazards regression model was fitted using the “coxph” function from the survival package v3.2–13 on the survival object produced by the “Surv” function. To aid in model selection, we performed univariate Cox regression on the following variables: age, sex (male), diabetes status, systolic blood pressure, BMI, LDL-cholesterol, HDL-cholesterol, and triglycerides levels, C-reactive protein (CRP) values, LVEF, NT-proBNP levels, kidney function, smoking status, the use of statins, ethnicity, and ImP quartiles. Dummy variables were used in lieu of categorical variables. Multivariate cox regression was fitted including all variables in 1 model and all but ImP-quartile in another. Resampling validation of the fitted model’s indexes of fit was performed using the “validate” function on the rms produced Cox proportional hazards model object (rms v6.2–0) using bootstrap with 1,000 iterations and with backward step-down variable deletion using Akaike’s information criterion as a stopping rule. Forest Plot for Cox proportional hazards model was produced using survminer version 0.4.9.

Data are represented as boxplots: middle line is the median, the lower and upper hinges are the first and third quartiles, the upper whisker extends from the hinge to the largest value no further than 1.5 × the IQR from the hinge and the lower whisker extends from the hinge to the smallest value at most 1.5 × IQR of the hinge.

## RESULTS

### IMIDAZOLE PROPIONATE IS INCREASED IN PATIENTS WITH CARDIOVASCULAR DISEASE AND HEART FAILURE IN THE EUROPEAN COHORT.

We previously observed that ImP levels are associated with prediabetes and T2D in a multicentric European (MetaCardis)^31^ cohort (n = 1,985). Here, we explored if serum concentrations of ImP are independently associated with CVD and HF. Patients with CVD and HF in MetaCardis were slightly older, with a larger proportion of male patients, lower BMI, and impaired metabolic profile (increased glucose, insulin, and HbA_1c_) compared with subjects without CVD or HF (No CVD/HF) (Table 1). Subjects without CVD were primarily recruited from outpatient metabolic disease clinics, in which obesity and T2D are highly prevalent. Stable patients with CVD and HF were recruited from outpatient cardiology clinics, and, thus, individuals without CVD compared with those with CVD and HF displayed worse metabolic profiles.

We next stratified our study population according to the presence of HF and found that patients with HF had significantly higher serum levels of ImP compared with individuals with CVD and those without CVD or HF: *P* < 0.01 and *P* < 0.001, respectively (Figure 1A). When stratifying the population into quartiles of ImP, we observed that—after adjusting for traditional risk factors and other baseline covariates including kidney function—individuals in the highest quartile were associated with a higher likelihood of having HF compared with those in the lowest quartile (adjusted OR: 3.02 [95% CI: 1.17–7.72]; *P* < 0.05) (Supplemental Table 1).

To further characterize the association between ImP and indices of left ventricular systolic function, we stratified the study population according to LVEF and observed significantly higher levels of ImP in individuals with reduced LVEF (adjusted *P* < 0.001) (Figure 1B). Moreover, serum levels of amino-terminal proANP and NT-proBNP, serum biomarkers of cardiomyocyte stress or strain,^46^ were significantly increased with increasing ImP quartiles: adjusted *P* < 0.001 (Supplemental Figure 1).

### IMIDAZOLE PROPIONATE IS ASSOCIATED WITH CVD AND HF IN AN INDEPENDENT NORTH AMERICAN VALIDATION COHORT.

**FIGURE 2:**
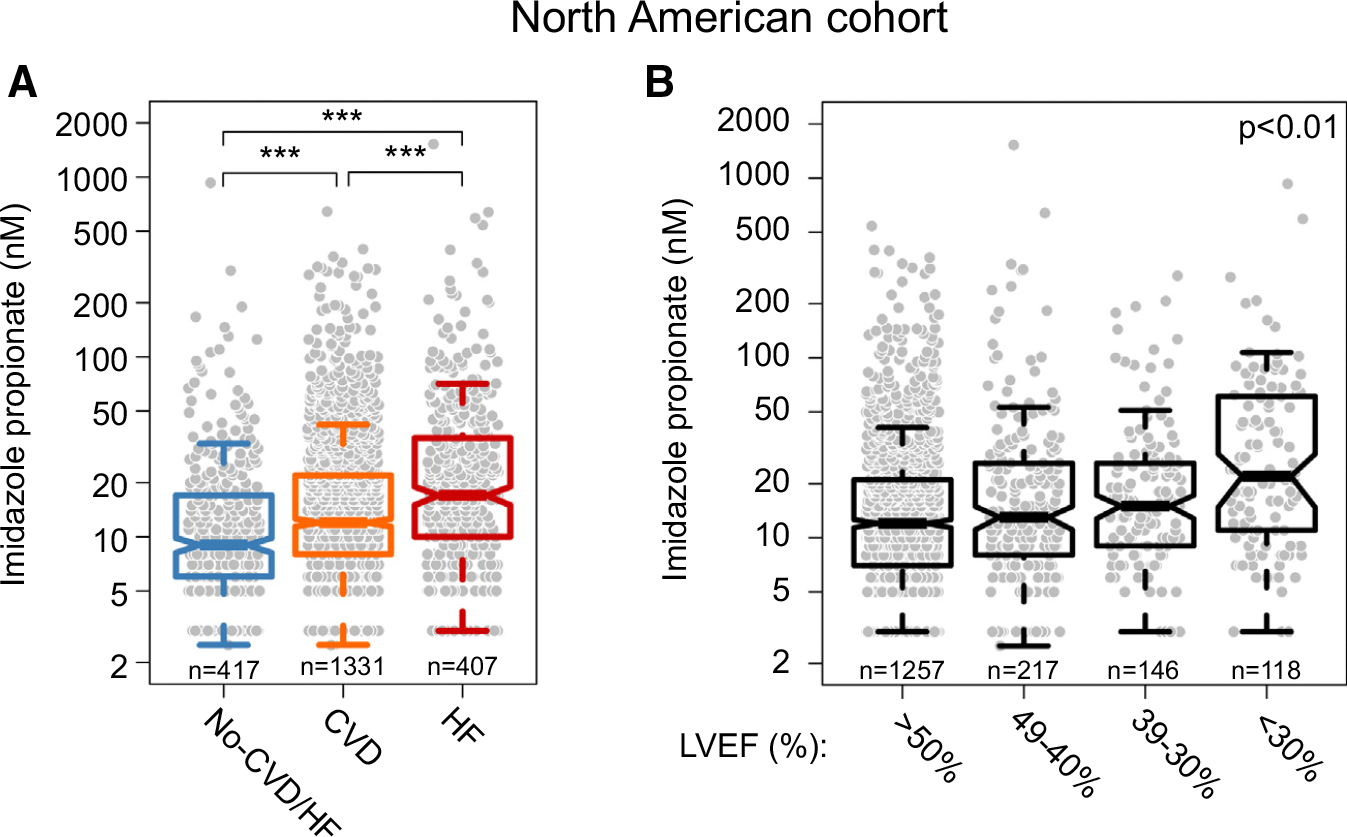
Imidazole Propionate in Individuals With Cardiovascular Diseases in the North American Cohort **(A)** Serum levels of imidazole propionate in individuals without CVD and HF (n = 417), with CVD (n = 1,331), and with HF (n = 407) (*P* values were calculated using Kruskal-Wallis H test, ***P < 0.001). **(B)** Serum levels of imidazole propionate according to percentage of LVEF (*P* values were calculated with linear regression model after adjustment for risk factors and other baseline covariates: age, sex, BMI, ethnicity, diabetes status, smoking status, systolic blood pressure, use of statins, circulating levels of HDL, LDL, triglycerides, and eGFR). Abbreviations as in Figure 1.

We next sought to determine whether the strong association between ImP levels and CVD and HF risks observed in the multicenter European Cohort could be replicated in an independent validation cohort. The association between ImP levels and both CVD and HF were thus first examined in a subset of GeneBank, a North American cohort composed of sequentially recruited stable patients undergoing elective diagnostic coronary evaluation at a tertiary referral center (n = 2,155).^8,47^ The baseline characteristics of the participants are shown in Table 2. We first confirmed our previous findings^30,31^ that circulating levels of ImP are associated with T2D (*P* < 0.01) (Supplemental Figure 2A). We then stratified the North American study population according to the presence of CVD and HF and observed that both patients with CVD and HF had significantly higher serum levels of ImP compared with individuals without CVD and HF (both comparisons *P* < 0.001) (Figure 2A). When stratifying the population in quartiles of ImP, we observed that persons in the highest quartile had a significantly higher likelihood of having HF compared with those in the lowest quartile, including after adjusting for traditional risk factors and other baseline covariates, as stated here (adjusted OR: 2.89 [95% CI: 1.79–4.66]; *P* < 0.001) (Supplemental Table 2).

In further analyses, the study population was stratified by LVEF (similar to analyses performed within the European cohort) to evaluate the impact of ImP on systolic function. We again observed that higher levels of ImP were observed in individuals with reduced LVEF: adjusted *P* < 0.01 (Figure 2B). Moreover, circulating levels of NT-proBNP were significantly increased with increasing ImP quartiles: adjusted *P* < 0.0001 (Supplemental Figure 2C). Taken together, our observations from 2 independent cohorts suggest that ImP levels are a significant independent predictor for the presence of CVD, the presence of HF, and 2 distinct HF-associated phenotypes (both impaired left ventricular systolic function and heightened circulating levels of natriuretic peptide levels).

### ImP IS ASSOCIATED WITH ALL-CAUSE MORTALITY.

**FIGURE 3:**
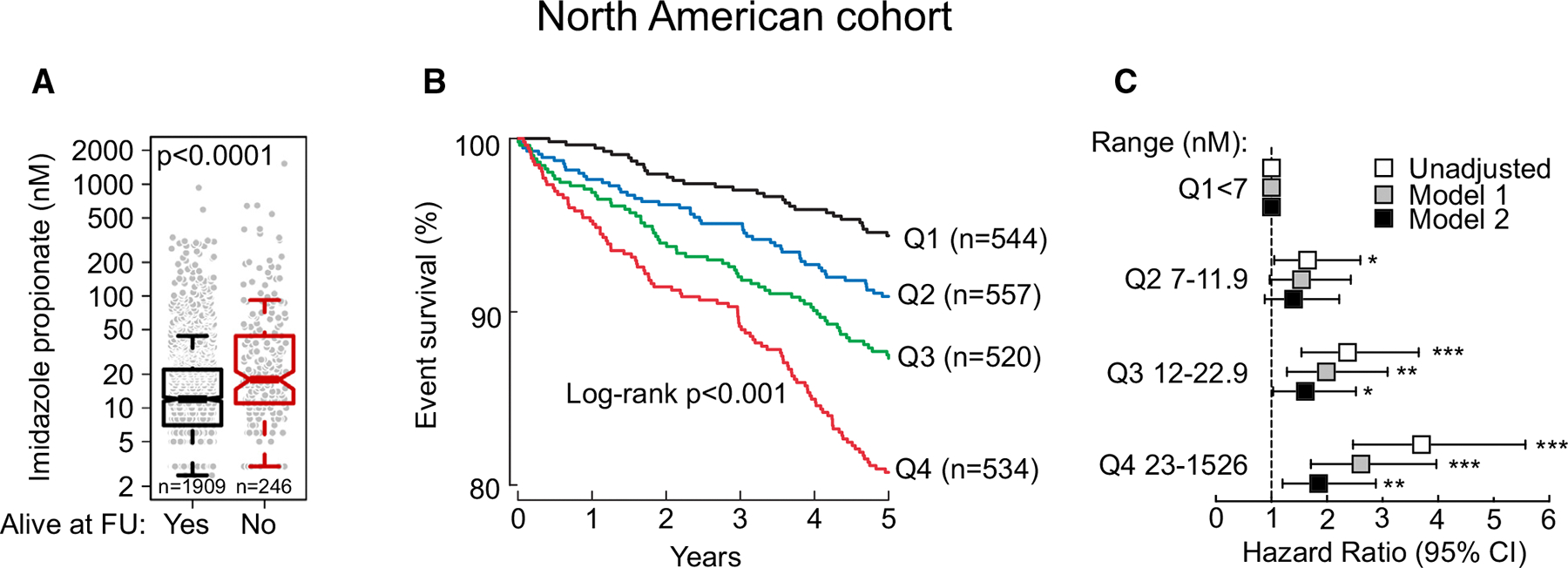
Serum Imidazole Propionate Levels Affect Survival in the North American Cohort **(A)** Serum levels of imidazole propionate levels among patients alive (Yes; n = 1,909) or not (No; n = 246) at 5-year follow-up (FU). *P* values were calculated using Mann-Whitney-U-test). Kaplan-Meier estimates **(B)** and Forrest plot **(C)** the risk of all-cause mortality at 5-year follow-up according to quartiles (Q) of imidazole propionate. The 5% to 95% CI is indicated by line length. *P < 0.05, **P < 0.01, ***P < 0.001. See Supplemental Table 5.

Next, we assessed if ImP levels in the North American cohort were associated with all-cause mortality during 5 years of follow-up and observed that higher baseline circulating ImP levels were associated with poorer survival (*P* < 0.0001, Figure 3A; and *P* < 0.001, Figure 3B). In agreement with these findings, we observed that high levels of ImP (Q4) were independently associated with an increased risk of overall mortality after adjusting for traditional risk factors and other baseline covariates (adjusted HR: 1.85 [95% CI: 1.20–2.88]; *P* < 0.01) (Figure 3C, Supplemental Table 3).

To elucidate if ImP levels associate with mortality independent of other pertinent predictors at 5 years, we performed Cox regression using traditional cardiovascular risk factors and other potentially relevant available covariates. To aid model selection, we performed univariate Cox regression for the following variables (as described in Methods). Each of these factors was assessed through a separate univariate Cox regression, which yielded statistically significant coefficients for age, diabetes status, creatinine, hypertension, LVEF category and ImP quartiles as well as BMI, CRP, NT-proBNP, and HDL-cholesterol levels, albeit with very low effect size for the last 4 variables (Supplemental Table 4, Supplemental Figure 3). Subsequently, we fitted a multivariate Cox regression including all variables in 1 model and all but ImP quartile in another. Analysis of variance (ANOVA) of the 2 models yielded a chi-square value of 5.72 and value of *P* = 0.016: concordance = 0.749 (SE = 0.018) without and concordance = 0.755 (SE = 0.017) with ImP as an added variable, and after inclusion of ImP quartile, only age, NT-proBNP, LVEF, smoking, and ImP remained significant (HR_ImP_ 1.18, value of *P* < 0.05). We further conducted resampling validation of the fitted model’s indexes with bootstrap (1,000 iterations) and included an automatic factor selection. Factors retained in backward elimination, and, hence, included in the final model were age, diabetes status, triglyceride levels, LVEF, NT-proBNP levels, smoking status, and ImP quartile (Supplemental Table 5), suggesting that, beyond known cardiovascular risk factors, ImP is an independent predictor for 5-year mortality.

## DISCUSSION

Patients with T2D have more than twice the risk of developing HF than those without T2D.^48,49^ Despite the implementation of multifactorial T2D treatment regimens, including glucose-lowering, blood-pressure–lowering, and lipid-lowering medication, the high prevalence of CVD and HF persists. This raises the possibility that additional factors beyond glycemia might contribute to the increased HF risk in T2D. Whether these additional factors for both T2D and CVD (with and without HF) might serve as targets for therapeutic interventions to reduce the risk of HF and adverse CVD outcomes is unknown. We have recently identified a novel gut microbial pathway enriched in T2D that includes ImP production from the amino acid histidine.^30,31,35^ Here, we demonstrate that ImP levels were associated with CVD and HF in 2 large independent cohorts, independently of T2D and other established cardiovascular risk factors. Furthermore, we showed that ImP levels are an independent risk factor for overall mortality after adjustment for known risk factors. Whether these clinical associations are observed because of some underlying causal contribution between ImP and CVD or HF pathogenesis remains unknown.

HF is a multifactorial disease and microbiota and microbially produced metabolites may be overlooked factors that can contribute to pathogenesis of disease in specific subpopulations.^50^ The HF-associated microbiota is similar to that observed in other metabolic diseases with reduced bacterial diversity and reduced abundance of butyrate producing bacteria such as *Faecalibacterium prausnitzii.*^30,31,51^ In previous studies, we identified bacterial taxa associated with ImP production (eg, *Streptococcus mutans, Eggerthella lenta, Clostridium symbiosum, Pseudoflavonifractor, Eubacterium eligens*)^30,31^ that are also enriched in patients with HF in the European cohort.^51^ ImP induces insulin resistance in mouse models by inducing mTORC1 signaling through p38γ/δ^30^, and mice lacking p38γ/δ do not develop heart hypertrophy.^39,52^ In addition, ImP inhibits AMPK signaling, which is a key sensor of cellular energy.^35^ As both pathways have been linked with cardiac fibrosis, hypertrophy, and HF,^53,54^ ImP may contribute to HF through these mechanisms. However, further studies, using ImP long-term delivery in animal models of HF, are required to clarify if and how ImP affects HF pathogenesis directly.

### STUDY STRENGTHS.

The major strengths are that we use large independent cohorts from 2 continents and—using quantitative stable isotope dilution LC-MS/MS methodology—identify increased ImP to be associated with risk of CVD and HF. Our study includes different CVD- and HF-relevant phenotypes, and ImP shows associations with mortality independent of traditional risk factors in both cohorts with all clinical and phenotypic assessments. Finally, cross-sectional data showed consistent associations between cohorts.

### STUDY LIMITATIONS.

Only a single time point is available in each cohort for the assessment of ImP levels. Moreover, only fasting levels are available, although it remains unclear whether unfasted levels are more relevant for outcome predictions for this gut-microbiota–dependent metabolite. It should also be noted that the European and North American cohorts differ in terms comorbidities and risk factors that might influence ImP levels (eg, age, presence of metabolic diseases, and both prevalence and severity of HF within the cohort). These differences likely contribute to the smaller differences in ImP levels between control subjects and patients with HF within the European cohort compared with the North American cohort. However, despite this limitation, we could observe significant consistent differences in ImP levels between individuals without or with HF in both cohorts. Further studies are required to investigate how ImP affects HF etiology in different patient subgroups (eg, ischemic vs nonischemic and HFpEF vs HFmEF vs HFrEF). Finally, our study was not designed to address if ImP levels predicted progressive worsening of symptoms, increased HF hospitalizations, or need for transplantation.

## CONCLUSIONS

Here, we showed that ImP, which is produced by the gut microbiota,^30^ was associated with reduced LVEF and HF in 2 large clinical cohorts from different continents (Central Illustration). As gut-microbiota compositions differ among countries,^55^ regions,^56^ and even among ethnicities within a city,^57^ circulating microbially produced metabolites, which are independent of the microbial taxonomy, may provide stronger and more generalizable findings. The metabolites may also provide mechanistic insights between altered intestinal microbiota and cardiometabolic diseases.^2,50,58^ Importantly, we found that the association among ImP and CVD and HF was independent of obesity and T2D, as both obesity and T2D are associated with progression of disease.^59^ In conclusion, our data suggest a strong association among ImP levels and CVD, HF, and HF-associated phenotypes including reduced left ventricular systolic ejection fraction, and elevated natriuretic peptide levels, independent of traditional CVD risk factors.

## Supplementary Material

1

**APPENDIX** For supplemental tables and figures, please see the online version of this paper.

## Figures and Tables

**TABLE 1:** Characteristics of the European Cohort

	Total (N = 1,984)	No-CVD/HF (n = 1,569)	CVD (n = 282)	HF (n = 133)	*P* Value

Age, y	56.45 ± 12.3	55.2 ± 12.8	61 ± 8.7	61.4 ± 9.9	<0.001
Male	50.5	41.9	84.6	78.0	<0.001
Caucasian ethnicity	86.3	86.4	85.5	84.7	NS
BMI, kg/m^2^	30.5 (25.8–37.6)	32.1 (26.3–39.5)	27.4 (24.8–30.5)	28.2 (24.7–32.5)	<0.001
Glucose, mmol/L	5.7 (5.3–6.3)	5.7 (5.1–7)	5.6 (5.2–6.3)	5.7 (5.1–7.1)	<0.001
HbA_1c_	5.7 (5.1–6.9)	5.8 (5.5–6.6)	5.6 (5.2–6.3)	6.1 (5.7–6.7)	NS
LDL-c, mmol/L	2.8 (2.2–3.5)	3.0 (2.5–3.7)	1.9 (1.5–2.5)	2.3 (1.8–2.7)	<0.001
HDL-c, mmol/L	1.2 (1–1.5)	1.3 (1.1–1.6)	1.1 (0.9–1.3)	1.1 (0.9–1.3)	<0.001
Triglycerides, mmol/L	1.3 (0.9–1.8)	1.3 (0.9–1.8)	1.2 (0.8–1.6)	1.3 (0.9–1.6)	NS
Creatinine, μmol/L	76.0 (66.0–87.0)	73.0 (64.0–84.0)	82.0 (73.0–93.0)	87.0 (75.0–106.0)	<0.001
Hypertension	74.8	70.2	89.7	96.5	<0.001
Systolic BP, mm Hg	129 (119–140)	130 (119–141)	125 (116–134)	124 (114–137)	<0.001
Diabetes	38.6	40.7	29.8	32.6	<0.001
Smoking	13.7	12.6	18.2	18.2	<0.050
Statins	36.1	22.8	80.7	72.7	<0.001

Values are mean ± SD, %, or median (IQR). Characteristics of participants were compared across groups using linear regression for continuous variables and Fisher test for categorical variables.

BMI = body mass index; BP = blood pressure; CVD = cardiovascular disease; HbA_1c_ = glycosylated hemoglobin; HDL-c = high-density lipoprotein cholesterol; HF = heart failure; LDL-c = low-density lipoprotein cholesterol; NS = not significant.

**TABLE 2:** Characteristics of the North American Cohort

	Total (N = 2,155)	No-CVD/HF (n = 417)	CVD (n = 1,331)	HF (n = 407)	*P* Value

Age, y	62.9 ± 10.9	57.9 ± 9.4	63.5 ± 11	66.4 ± 10.3	<0.001
Male	64.1	44.8	71.4	60	<0.001
Caucasian ethnicity	95.5	92.8	96.3	95.3	<0.050
BMI, kg/m^2^	28.4 (25.5–32.2)	28.9 (25.3–33.3)	28.3 (25.6–31.9)	28.4 (25.2–32.9)	NS
Glucose, mmol/L	5.6 (5.1–6.2)	5.5 (5–6)	5.6 (5.3–6.1)	5.8 (5.4–6.4)	<0.001
HbA_1c_	5.7 (5.3–6.1)	5.5 (5.2–6.0)	5.7 (5.3–6.1)	5.8 (5.4–6.4)	<0.001
LDL-c, mmol/L	2.5 (2.0–3.0)	2.8 (2.3–3.3)	2.4 (1.9–3)	2.4 (1.9–2.8)	<0.001
HDL-c, mmol/L	0.9 (0.7–1.1)	1.0 (0.8–1.2)	0.9 (0.7–1)	0.8 (0.7–1)	<0.001
Triglycerides, mmol/L	1.3 (1.0–1.8)	1.1 (0.9–1.7)	1.4 (1–1.9)	1.3 (1–1.2)	<0.010
Creatinine, μmol/L	76.9 (67.2–89.3)	71.6 (62.8–81.4)	77 (67.3–88.5)	84.1 (71.7–103.5)	<0.001
Hypertension	70.5	52.9	74.5	75.5	<0.001
Systolic BP, mm Hg	132 (119–146)	133 (120–146)	133 (120–147)	127 (114–142)	<0.001
Diabetes	22.1	16.3	21.7	29.5	<0.001
Smoking	12.8	8.2	14.5	12.1	<0.010
Statins	59.1	30.9	66.9	62.4	<0.001

Values are mean ± SD, %, or median (IQR). Characteristics of participants were compared across groups using linear regression for continuous variables and Fisher test for categorical variables.

Abbreviations as in Table 1.

## PERSPECTIVES

### COMPETENCY IN MEDICAL KNOWLEDGE:

In cohort studies of adults from Europe and North America, high serum levels of imidazole propionate are associated with the presence of cardiovascular diseases and higher risks of cardiovascular events and mortality.

### TRANSLATIONAL OUTLOOK:

Further studies are needed to elucidate the direct mechanisms linking imidazole propionate with pathophysiology of cardiovascular disease onset and deterioration and cardiovascular outcomes.

## ABBREVIATIONS AND ACRONYMS

BMI: body mass index
CVD: cardiovascular disease
eGFR: estimated glomerular filtration rate
HDL: high-density lipoprotein
HF: heart failure
ImP: imidazole propionate
LDL: low-density lipoprotein
T2D: type 2 diabetes
TMAO: trimethylamine N-oxide
