# Supplementary material for: Microbially Produced Imidazole Propionate Is Associated With Heart Failure and Mortality
Source: JACC Heart Fail. Author manuscript; Available in PMC 2025 Oct 10. (PMC12512386; doi:10.1016/j.jchf.2023.03.008)
Supplement: 1 [file NIHMS2112556-supplement-1.pdf]

**Supplemental figures and tables.**

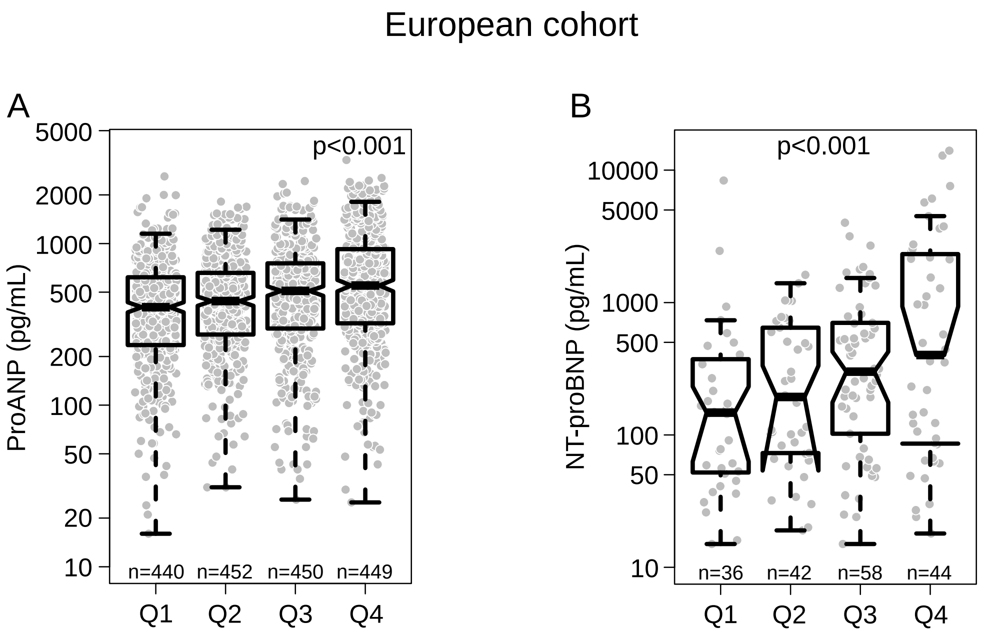


**Supplemental Figure 1. Imidazole propionate is associated with serum markers of reduced heart function in the European cohort. A** Serum levels of ProANP and NT-proBNP (**B**) according to quartiles of imidazole propionate (p values were calculated with linear regression model after adjustment for risk factors and other baseline covariates: age, sex, BMI, ethnicity, diabetes status, smoking status, systolic blood pressure, use of statins, serum levels of HDL, LDL, triglycerides and eGFR).

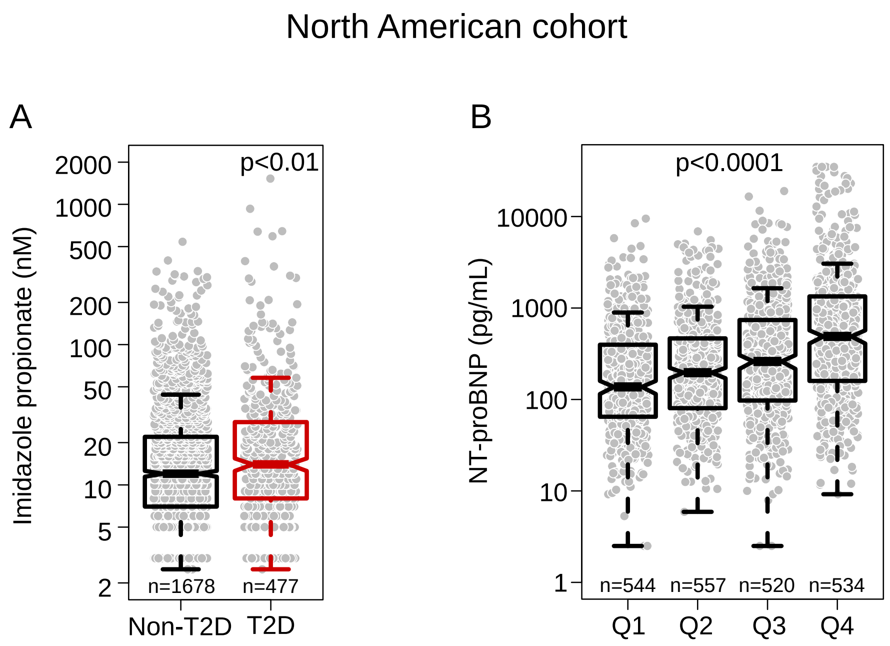


**Supplemental Figure 2. Imidazole propionate is increased in presence of type 2 diabetes and is associated with circulating markers of reduced heart function in the North American cohort. A.** Plasma levels of imidazole propionate in patients without (no-T2D; n=1678) and with (T2D; n=477) type 2 diabetes (p values were calculated using Mann-Whitney-U-test). **B.** Plasma levels of NT-proBNP according to quartiles of imidazole propionate (p values were calculated with linear regression model after adjustment for risk factors and other baseline covariates: age, sex, BMI, ethnicity, diabetes status, smoking status, systolic blood pressure, use of statins, circulating levels of HDL, LDL, triglycerides and eGFR).

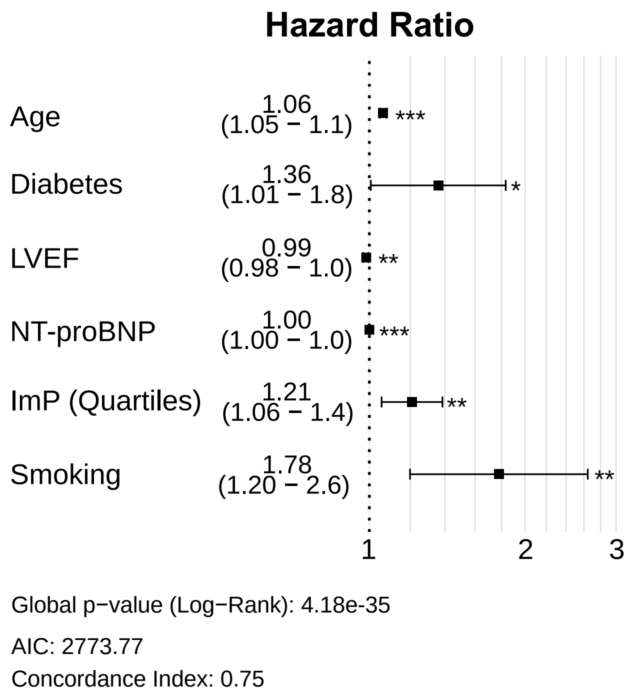


**Supplemental Figure 3. Imidazole propionate is a significant independent predictor for increased mortality at 5 years in the North American cohort.** Forest plot for Cox proportional hazards model showing the hazard ratio for mortality at 5 years for all significant variables derived from model selection after resampling validation. The model was built using data from 2154 subjects with available survival data (Number of events= 199). The 5–95% confidence interval is indicated by line length. *p<0.05, **p<0.01, ***p<0.001. See Supplementary table 4 and 5.

| | | CVD | | HF | |
|---|---|---|---|---|---|
| | Quartiles of ImP | Odds ratio (95% CI) | Odds ratio (95% CI) | Odds ratio (95% CI) | p value |
| **Unadjusted** | Q1 (<16.2) | reference | reference | reference | - |
| | Q2 (13.3-27.2) | 1.72 (1.17-2.53) | 1.72 (1.17-2.53) | 1.51 (0.73-3.11) | ns |
| | Q3 (27.3-52.5) | 1.98 (1.35-2.90) | 1.98 (1.35-2.90) | 3.97 (2.10-7.48) | <0.001 |
| | Q4 (52.7-901.2) | 1.94 (1.32-2.86) | 1.94 (1.32-2.86) | 5.40 (2.91-10.02) | <0.001 |
| **Model 1** | Q1 (<16.2) | reference | reference | reference | - |
| | Q2 (13.3-27.2) | 0.98 (0.47-2.02) | 0.98 (0.47-2.02) | 1.12 (0.43-2.88) | ns |
| | Q3 (27.3-52.5) | 1.15 (0.56-2.35) | 1.15 (0.56-2.35) | 2.47 (1.03-5.96) | <0.01 |
| | Q4 (52.7-901.2) | 1.05 (0.48-2.26) | 1.05 (0.48-2.26) | 3.78 (1.54-9.99) | <0.01 |
| **Model 2** | Q1 (<16.2) | reference | reference | reference | - |
| | Q2 (13.3-27.2) | 0.93 (0.51-1.69) | 0.93 (0.51-1.69) | 1.05 (0.40-2.80) | ns |
| | Q3 (27.3-52.5) | 1.09 (0.53-2.24) | 1.09 (0.53-2.24) | 2.25 (0.91-5.55) | ns |
| | Q4 (52.7-901.2) | 1.03 (0.47-2.25) | 1.03 (0.47-2.25) | 3.02 (1.17-7.72) | <0.05 |

**Table S1. Multinominal logistic regression for cardiovascular diseases (CVD) and heart failure (HF) in the European cohort.** Model 1 adjusted for age, sex, BMI, ethnicity, diabetes status, systolic blood pressure, smoking status, LDL, HDL, triglycerides, statin usage. Model 2 adjusted for model 1 plus estimated glomerular filtration rate. ImP, Imidazole propionate; C.I., confidence interval; Q, quartile.

| | | CVD | | HF | |
|---|---|---|---|---|---|
| | **Quartiles of ImP** | **Odds ratio (95% CI)** | **p value** | **Odds ratio (95% CI)** | **p value** |
| **Unadjusted** | Q1 (<7) | reference | - | reference | - |
| | Q2 (7-12) | 1.63 (1.22-2.17) | <0.01 | 1.73 (1.15-2.60) | <0.01 |
| | Q3 (12-23) | 2 (1.47-2.72) | <0.001 | 3.09 (2.06-4.65) | <0.001 |
| | Q4 (23-1526) | 2.34 (1.69-3.25) | <0.001 | 5.62 (3.74-8.45) | <0.001 |
| **Model 1** | Q1 (<7) | reference | - | reference | - |
| | Q2 (7-12) | 1.14 (0.83-1.58) | ns | 1.34 (0.86-2.11) | ns |
| | Q3 (12-23) | 1.58 (1.11--2.25) | <0.05 | 2.53 (1.61-3.98) | <0.001 |
| | Q4 (23-1526) | 1.65 (1.14-2.39) | <0.01 | 3.72 (2.36-5.86) | <0.001 |
| **Model 2** | Q1 (<7) | reference | - | reference | - |
| | Q2 (7-12) | 1.16 (0.84-1.62) | ns | 1.24 (0.79-1.95) | ns |
| | Q3 (12-23) | 1.65 (1.15-2.36) | <0.05 | 2.19 (1.38-3-47) | <0.001 |
| | Q4 (23-1526) | 1.78 (1.20-2.61) | <0.05 | 2.89 (1.79-4.66) | <0.001 |

**Table S2. Multinominal logistic regression for cardiovascular diseases (CVD) and heart failure (HF) in North American cohort**. Model 1 adjusted for age, sex, BMI, ethnicity, diabetes status, systolic blood pressure, smoking status, LDL, HDL, triglycerides, statin usage. Model 2 adjusted for model 1 plus estimated glomerular filtration rate. ImP, imidazole propionate; C.I., confidence interval; Q, quartile.

| | Quartiles of ImP | HR (95% CI) | p value |
|---|---|---|---|
| **Unadjusted** | Q1 (<7) | reference | - |
| | Q2 (7-12) | 1.65 (1.05-2.60) | <0.05 |
| | Q3 (12-23) | 2.37 (1.54-3.65) | <0.001 |
| | Q4 (23-1526) | 3.70 (2.47-5.57) | <0.001 |
| **Model 1** | Q1 (<7) | reference | - |
| | Q2 (7-12) | 1.54 (0.97-2.43) | ns |
| | Q3 (12-23) | 1.99 (1.28-3.09) | <0.01 |
| | Q4 (23-1526) | 2.61 (1.71-3.97) | <0.001 |
| **Model 2** | Q1 (<7) | reference | - |
| | Q2 (7-12) | 1.40 (0.88-2.22) | ns |
| | Q3 (12-23) | 1.61 (1.03-2.52) | <0.05 |
| | Q4 (23-1526) | 1.85 (1.20-2.88) | <0.01 |

**Table S3. Cox-regression models for overall mortality at 5 years follow-up in the North American cohort**. Model 1 adjusted for age, sex, BMI, ethnicity, diabetes status, systolic blood pressure, smoking status, LDL, HDL, triglycerides, statin usage. Model 2 adjusted for model 1 plus estimated glomerular filtration rate. ImP, imidazole propionate; HR, hazzard ratio; C.I., confidence interval; Q, quartile.

| | Beta-coefficient | HR (95% CI) | Wald test | p value |
|---|---|---|---|---|
| Age | 0.065 | 1.1 (1.1-1.1) | 98 | <0.001 |
| Male sex | -0.094 | 0.91 (0.7-1.2) | 0.51 | ns |
| Diabetes | 0.48 | 1.6 (1.2-2.1) | 12 | <0.001 |
| Systolic blood pressure | 0.0019 | 1 (1-1) | 0.37 | ns |
| BMI | -0.026 | 0.97 (0.95-1) | 4.7 | <0.05 |
| LDL-cholesterol levels | -0.0047 | 1 (0.99-1) | 5 | <0.05 |
| Triglyceride levels | 0.00068 | 1 (1-1) | 1.2 | ns |
| HDL-cholesterol levels | -0.017 | 0.98 (0.97-1) | 6.7 | <0.01 |
| hsCRP | 0.0097 | 1 (1-1) | 20 | <0.001 |
| LVEF | -0.03 | 0.97 (0.96-0.98) | 42 | <0.001 |
| LVEF (categories) | 0.36 | 1.4 (1.3-1.6) | 32 | <0.001 |
| NT-proBNP | 1.00E-04 | 1 (1-1) | 130 | <0.001 |
| Statin usage | -0.062 | 0.94 (0.73-1.2) | 0.23 | ns |
| eGFR | 0.3 | 1.4 (1.2-1.5) | 56 | <0.001 |
| ImP (quartiles) | 0.42 | 1.5 (1.4-1.7) | 48 | <0.001 |
| Hypertension | 0.31 | 1.4 (1-1.8) | 4.2 | <0.05 |
| Smoking | 0.14 | 1.2 (0.81-1.6) | 0.61 | ns |
| Caucasian ethicity | -0.28 | 0.75 (0.44-1.3) | 1.1 | ns |

**Table S4. Univariate factors Cox proportional-hazards regression for all-cause mortality at 5 years in the North American cohort.** Each factor is assessed through separate univariate Cox regressions. BMI, body mass index; HDL, high density lipoprotein; LDL low density lipoprotein; eGFR, estimated glomerular filtration rate; LVEF, left ventricular ejection fraction (≥50%, 49-40%, 39-30%. <30%); NT-ProBNP, N-terminal prohormone of brain natriuretic peptide; ImP: imidazole propionate; HR, hazzard ratio; CI, confidence interval.

| | Coefficient | Standard error | Wald Z | p-value |
|---|---|---|---|---|
| Age | 6.426e-02 | 7.666e-03 | 8.382 | 0.000e+00 |
| Diabetes | 2.947e-01 | 1.572e-01 | 1.875 | 6.086e-02 |
| Triglyceride levels | 1.557e-03 | 6.989e-04 | 2.228 | 2.589e-02 |
| LVEF | -1.372e-02 | 5.185e-03 | 2.647 | 8.124e-03 |
| NT-ProBNP | 7.702e-05 | 1.245e-05 | 6.187 | 6.145e-10 |
| Smoking | 5.774e-01 | 2.050e-01 | 2.817 | 4.853e-03 |
| ImP (Quartiles) | 1.872e-01 | 6.996e-02 | 2.676 | 7.444e-03 |

| | Index Orig | Training | Test | Optimism | Index corrected | n |
|---|---|---|---|---|---|---|
| Somer's D (Dxy) | 0.5055 | 0.5239 | 0.4869 | 0.037 | 0.4685 | 1000 |
| Nagelkerke's R2 | 0.1217 | 0.1349 | 0.1119 | 0.0230 | 0.0987 | 1000 |
| Calibration slope | 1.000 | 1.000 | 0.8808 | 0.1192 | 0.8808 | 1000 |
| Discrimination index (D) | 0.0611 | 0.0682 | 0.559 | 0.0123 | 0.0487 | 1000 |
| Unreliability index (U) | -0.0007 | -0.0007 | 0.0030 | -0.0037 | 0.0030 | 1000 |
| Overall quality index (Q) | 0.0618 | 0.0689 | 0.0529 | 0.0160 | 0.0457 | 1000 |
| g-Index | 1.0221 | 1.0893 | 0.9548 | 0.1345 | 0.8876 | 1000 |

**Table S5. Approximate coefficients after factor deletion for remaining significant factors in the final model and validation estimates for the final model (bootstrap with 1000 iterations).** LVEF, left ventricular ejection fraction; NT-ProBNP, N-terminal prohormone of brain natriuretic peptide; ImP: Imidazole Propionate.
